# A Study in Economic Medicine

**Published:** 1910-11-26

**Authors:** 


### The Hospital

A Journal of

The Medical Sciences and Hospital Administration.

New Series. No. 198, Vol. VIII. [No. 1270, Vol. XLIX.] SATURDAY, NOVEMBER 26, 1910.

### A Study in Economic Medicine.

The conquest of the tropics is not yet accomplished, though the ramparts have been breached and forlorn hopes of stormers have here and there made good their footing within the walls. The campaign so far has been very arduous, although it has only just begun; but the attacking forces have been heartened by a series of brilliant victories in the preliminary skirmishes, and the ultimate result must be a complete victory. To the long list of leaders in the fray the gratitude of the whole world is due, though many of them—most of them, in fact—will never be known beyond a limited circle of experts. There are, however, some of the greatest whom an enlightened posterity will honour when the politicians and soldiers of the present era are all forgotten. Of such are Laveran, Manson, Ronald Ross. Without undervaluing the work of the two former, or of any other toiler in the field of malaria research, to the last mentioned belongs the honour of establishing the truth of the mosquito theory of malaria, and to him more than to any other one man the world assigns the credit of showing how the deadly tropics may be rendered habitable.

What Clausewitz and Mahan are to army and navy staff officers, that Major Ronald Ross, through his lately published book,* is to tropical hygienists. Rarely indeed has one the pleasure of reading so masterly an analysis as this of such an intricate and technical subject. Whoso reads this book must realise that it has been by no fluke that Major Ross has made the discoveries for which he is famous. Explained in such language that any well-educated and intelligent man can grasp the sequence of the argument, the marshalling of facts and figures displays a precision which is evidence of the logical mind and fertile brain necessary to a great philosopher. Major Ross is eminently a strategist and a tactician, as well as a general. He has, it is true, one advantage over the commander of an army in the field, in that his enemy's methods are always the same, and are capable of mathematical analysis; but this is offset by the fact that he has had to puzzle out for himself the very rudiments of his plans of campaign, to train his own raw recruits as it were. His work has already been the means of saving hundreds of thousands, probably several millions, of human lives, and unquestionably will save those of unborn myriads. Yet, as far as public official recognition is concerned, this winner of a Nobel prize eight years ago remains but a C.B., thus classed with the more incompetent brigadiers and barely average regimental commanders in the South African war. Services rendered to a party one hundredth part as valuable as those which Major Ross has rendered to the human race would have received a far more substantial recognition!

So vastly important is the prevention of malaria that no measure which can spread enlightened knowledge about it can be regarded as unnecessary. Speaking in all seriousness and with the profoundest conviction, we would advocate the addition of a paper in this subject to the examination of candidates for the Indian Civil Service. If the clever and highly educated young men who enter for that examination found that by mastering this book they could obtain two or three hundred marks in the competition, it is perfectly certain that India would soon be administered by civil officials who could be relied on to afford expert and sympathetic assistance to the anti-malarial crusade. At the same time, the candidates themselves would obtain a training in scientific method which could not fail to benefit them all immensely.

To review this book in any detail is impossible, for every section teems with ideas and with instruction. If a slightly egotistical note is struck in the first chapter that is perhaps not very astonishing; if a dogmatical tendency exists here and there—after all, earnestness and thoroughness go with it always. Rightly contemptuous is Major Ross of the spirit of Indian fakirism, as he terms a tendency of the age, according to which all matters of practical utility to mankind are looked upon as being rather base occupations for the more perfect type of human being. "The scientific side of administration is apt to be forgotten in the noise

* *The Prevention of Malaria.* By Ronald Ross. London: John Murray, 1910. Pp. 669. Price 21s. net.

of endless and despicable party strife; progress ceases while we discuss abstract notions about law, liberty, representation, nationalism, and so forth; the machine refuses to work while the mechanics are quarrelling over the lubrication. The result is precisely what may be imagined—cities built without sense or forethought, filthy slums, hovels filled with disease, gulfs of destitution; and the voices of those who would better this state of affairs by scientific methods are lost among the yells of opposing factions." Inaction and irrationalism are faults of Governments which it will take centuries to eradicate; but at least there is less excuse than ever for them in respect to malaria prevention since the appearance of this book.

Whether it is in the higher mathematics of endemiology, or the practical details of personal mosquito evasion, or patient laboratory research, or in the discussion of difficult problems of economics, the author is equally at home. The lucidity of arrangement and expression are not the least among the virtues of this notable book, which is an encyclopædia of the anti-malaria measures of civilised society. After the 330 pages in which the author explains the problems presented and the best lines along which to solve them, the remainder of the book is taken up by the accounts of special contributors, from all over the world, of their own works and progress. This list of twenty contributors includes the leading workers in our Colonies and abroad, and their special experiences are described in reference to the local conditions which are met with in the various tropical countries where their lot has been cast. Examples of legislation and a good bibliography are included in appendices, and complete a book which takes immediate rank as a standard authority.

